# A Maluku Sea intermediate western boundary current connecting Pacific Ocean circulation to the Indonesian Throughflow

**DOI:** 10.1038/s41467-022-29617-6

**Published:** 2022-04-19

**Authors:** Dongliang Yuan, Xueli Yin, Xiang Li, Corry Corvianawatie, Zheng Wang, Yao Li, Ya Yang, Xiaoyue Hu, Jing Wang, Shuwen Tan, Dewi Surinati, Adi Purwandana, Adhitya Kusuma Wardana, Mochamad Furqon Azis Ismail, Asep Sandra Budiman, Ahmad Bayhaqi, Praditya Avianto, Priyadi Dwi Santoso, Edi Kusmanto, Dirhamsyah, Zainal Arifin, Larry J. Pratt

**Affiliations:** 1 Key Laboratory of Ocean Circulation and Waves, and Center for Ocean Mega-Science, Institute of Oceanology, Chinese Academy of Sciences, 7 Nanhai Road, Qingdao, China; 2 Key Laboratory of Marine Science and Numerical Modeling, First Institute of Oceanography, Ministry of Natural Resources, 6 Xianxialing Road, Laoshan District, Qingdao, China; 3 Pilot National Laboratory for Marine Science and Technology (Qingdao), Qingdao, China; 4 Shandong Key Laboratory of Marine Science and Numerical Modeling, 6 Xianxialing Road, Laoshan District, Qingdao, China; 5 University of Chinese Academy of Sciences, Beijing, China; 6 Research Center for Oceanography–National Research and Innovation Agency (RCO-BRIN), Jakarta, Indonesia; 7 Department of Physical Oceanography, Woods Hole Oceanographic Institution, Woods Hole, Falmouth, MA USA

**Keywords:** Physical oceanography, Physical oceanography

## Abstract

The Indonesian Throughflow plays an important role in the global ocean circulation and climate. Existing studies of the Indonesian Throughflow have focused on the Makassar Strait and the exit straits, where the upper thermocline currents carry North Pacific waters to the Indian Ocean. Here we show, using mooring observations, that a previous unknown intermediate western boundary current (with the core at ~1000 m depth) exists in the Maluku Sea, which transports intermediate waters (primarily the Antarctic Intermediate Water) from the Pacific into the Seram-Banda Seas through the Lifamatola Passage above the bottom overflow. Our results suggest the importance of the western boundary current in global ocean intermediate circulation and overturn. We anticipate that our study is the beginning of more extensive investigations of the intermediate circulation of the Indo-Pacific ocean in global overturn, which shall improve our understanding of ocean heat and CO_2_ storages significantly.

## Abstract (web summary)

Here the authors use in situ mooring data in the Maluku Channel and the Lifamatola Passage of the Indonesian seas to show that a western boundary current transports Antarctic water from the South Pacific to the Indian Ocean.

## Introduction

**Fig. 1:**
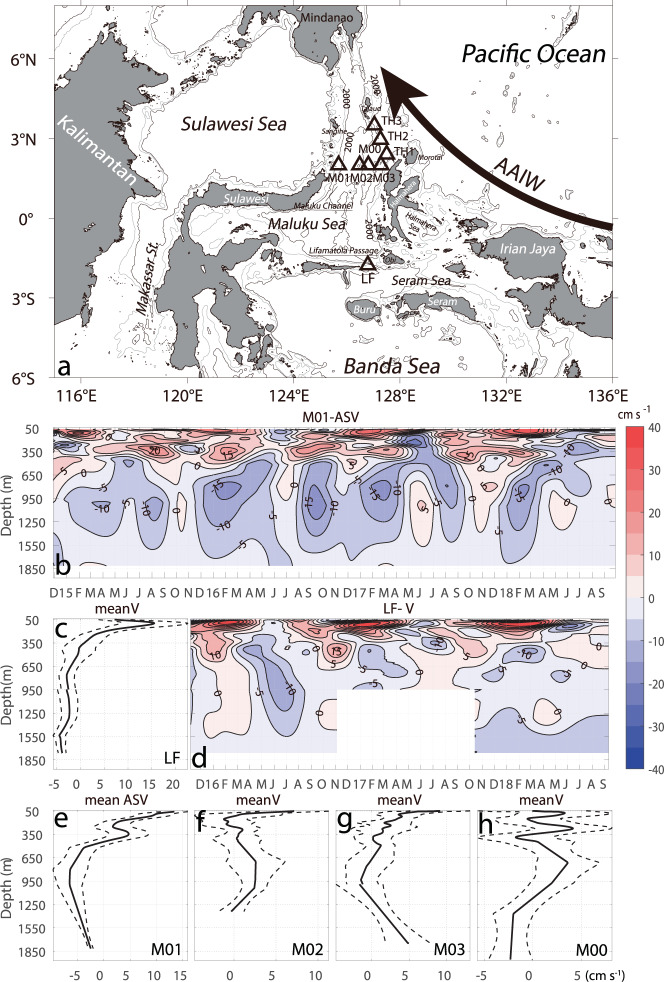
Mooring observations of Maluku Sea currents. **a** Map and locations of the eastern Indonesian sea moorings, with topography (unit in meters). The moorings from west to east are M01, M02, M00, M03 in the Maluku Channel. The big arrow in (**a**) mark the movement of the Antarctic intermediate water (AAIW) in the tropical western Pacific Ocean. 120-day low-passed daily mooring velocity at the sites of M01 and in the Lifamatola Passage (**b**, **d**), showing the mean intermediate depth western boundary current at the depth between 450 m and 1000 m (**e**–**h**) connecting to the southward flow at the same depth in the Lifamatola Passage (**c**). The along strait velocity (ASV) at M01 follow the coasts at an angle of 12° from due north. Only the meridional velocity is shown at M02, M00, and M03, due to lack of guidance by the coasts and the topography. The meridional velocity component is shown at the LF mooring, because the currents direction rotates significantly in the upper 1200 m, not following the thalweg in general. Dash lines mark the standard errors of the means at 95% significance. Contour interval is 5 cm s^−1^. The data blank in (**b**) is due to failure of an RCM at 1800 m from November 2016 to October 2017.

Observations have shown that the Antarctic Intermediate Water (AAIW) generated in the Sub-Antarctic Front zone of the Southern Ocean is transported into the equatorial western Pacific by the New Guinea Coastal Undercurrent (NGCUC)^1–4^, reaching as far north as east of the Mindanao Island^5^ (Fig. 1). The AAIW forms a low salinity intermediate layer in all of the southern hemisphere oceans north of the Antarctic Circumpolar Current, with potential temperature 4 °C < θ < 6 °C, salinity 34.1 psu<S < 34.5 psu, and potential density 27.05 kg m^−3^ < σ_θ_ < 27.15 kg m^−3^ in the southeast Pacific^6^. In the vicinity of the Pacific equatorial western boundary, it is identified by the salinity minimum (<34.5 psu) around the potential density of 27.2 σ_θ_^7,8^, centered at the depths of 600–900 m^8,9^. The salinity minimum is just the upper boundary of the AAIW layer. The layer down to ~27.5 σ_θ_ is generally identified as the AAIW, which covers thickness of several hundred meters^10^. Considering the strong mixing at the entrance of the Indonesian seas^9,11,12^, the part of the water masses below the North Pacific Intermediate Water (NPIW, with S < 34.45 psu, 26.2 kg m^−3^ < σ_θ_ < 26.9 kg m^−3^)^13^ and above the top of the Upper Circumpolar Deep Water (UCDW, 34.64 psu < S < 34.7 psu, 1.2 °C < θ < 2.2 °C, 27.7 kg m^−3^ < σ_θ_ <  27.98 kg m^−3^_θ_)^14^ is suggested to be dominated by the AAIW movement.

Existing studies of the Indonesian Throughflow (ITF) have focused on the currents in the Makassar Strait and the exit straits^15–17^. Due to the threshold depth as shallow as 680 m within the Makassar Strait, the AAIW in the western Pacific is not connected with the Indian Ocean circulation below this threshold through the Makassar Strait. In the eastern Indonesian seas, the sill depths of the Maluku Sea and Lifamatola Passage are as deep as 2000 m. Sparse historical hydrography measurements have shown a salinity minimum larger than that of the NPIW at about 300 m depths in the Maluku Channel, suggesting the intrusion of the lower part of the South Pacific Tropical Water (below the salinity maximum) into the Indonesian seas^15^. A salinity minimum layer at 600–700 m depths in the northern Maluku Sea, with the salinity minimum as high as 34.58 psu, might suggest the presence of AAIW mixed with the surrounding waters^18^. However, the path of the AAIW intrusion into the Indonesian seas has never been disclosed.

In this work, we identify a previous unknown intermediate western boundary current (WBC) in the Maluku Sea and through the Lifamatola Passage, using in situ mooring observations. The current carries the AAIW from the western equatorial Pacific into the Seram-Banda Seas between 450 m and 1800 m, and contributes about 1.36 Sv to the total ITF.

## Results

Maluku Channel mooring observations. Since 2014, a major Western Pacific Ocean Circulation-Indonesian Throughflow mooring array has been constructed by the Institute of Oceanology of the Chinese Academy of Sciences in the western Pacific and the Indonesian seas to measure the transports and water mass properties of the ITF, especially in the eastern route, in connection with the western Pacific and Indian Ocean circulation. The moorings inside the Indonesian seas were deployed and maintained using the Indonesian R/V Baruna Jaya VIII, in collaboration with the Research Center for Oceanography of Indonesian Institute of Sciences [now the National Research and Innovation Agency (BRIN)]. Three moorings (M01, M02, M03) were maintained in the Maluku Channel during November 2014 through November 2016 (Fig. 1). After November 2016, the section was occupied by two moorings at M01 and M00, with the latter being the deepest point of the eastern section. The moorings were equipped with one upward-looking ADCP at a nominal depth of 500 m and a few recorded current meters (RCMs) at different depths. The RCMs are either the Aanderaa acoustic current meters, manufactured by the Xylem Inc. of the USA, or the Aquadopp acoustic current meters of the Norway Nortek company. A few SBE37SM CTD instruments of the U.S. Sea-Bird Electronics Inc. are also attached to the mooring. We shall focus on the current meter data in this study. The configuration of these moorings is summarized in Table 1.

**Table 1:** Configurations of the moorings in the Maluku Channel, Lifamatola Passage, and the Talaud-Halmahera Channel.

Mooring	Longitude	Latitude	Deployment period	Deployment depth (m)	
				Current meter	ADCP (upward)
TH1	${127}^{o}{30.1}^{{\prime} }E$	${2}^{o}{24.1}^{{\prime} }{{{{{\rm{N}}}}}}$	2016.12–2018.09	750, 1000, 1800	450
TH2	${127}^{o}{16.0}^{{\prime} }E$	${2}^{o}{57.6}^{{\prime} }N$	2016.12–2018.09	750, 1000, 1800	450
TH3	${127}^{o}{2.3}^{{\prime} }E$	${3}^{o}{30.0}^{{\prime} }N$	2018.10–2020.01	750, 1000,1800	450
M01	${125}^{o}41.5^{\prime} E$	${2}^{o}N$	2014.11–2018.10	750, 1000, 1800	500
M02	${126}^{o}29.1^{\prime} E$	${2}^{o}N$	2014.11–2016.11	750, 1000, 1400	500
M00	${126}^{o}48.7^{\prime} E$	${2}^{o}N$	2016.11–2018.09	750, 1000, 1400, 2000	500
M03	${127}^{o}17.2^{\prime} E$	${2}^{o}N$	2014.11–2016.11	750, 1000, 1800	500
LF	${126}^{o}47.6^{\prime} E$	${1}^{o}45.7^{\prime} S$	2015.11–2017.10	750, 1000, 1800	500
			2017.10–2018.09	750,1000,1300	500, 1500(downward)

(1) No data at 1800 m during 2015.11–2016.11 at M03.

(2) No data at 1800 m during 2016.11–2017.10 at LF.

(3) No data at 1800 m during 2018.10–2020.01 at TH3.

At a point [126°47.6’E, 1°45.7’S] slightly upstream and west of the saddle point of the Lifamatola Passage, a mooring was maintained since November 2015. This mooring (called the LF mooring hereafter) was deployed in a water depth of 2100 m, located upstream of the historical mooring site of van Aken^19,20^. During the redeployment in October 2017, a downward looking ADCP was mounted at 1500 m to measure the bottom current profile (Table 1). The deep current that follows the topography is suggested to be sub-critical at the LF mooring site (slightly upstream of the sill), in contrast to the supercritical currents with a hydraulic jump downstream of the sill according to a recent analysis^21^.

The Maluku channel and the LF moorings form a set of synchronous current measurements at both of the entrance and exit of the southern Maluku Sea, which allow for examination of intermediate depth circulation and connections. In addition, three moorings were deployed in the Talaud-Halmahera Channel (hereafter called the TH Channel) during December 2016 and September 2018 to measure the exchange between the Maluku Sea and the western Pacific Ocean. Each mooring was equipped with an upward-looking ADCP at 450 m and a few RCMs at 250 m, 750 m, 1000 m, and 1800 m. Unfortunately, the lower part of the TH3 mooring was not recovered in the November 2017 cruise. Data of new deployment at the same site after October 2018 are used in the following analysis instead.

The mooring data at M01 have shown a persistent southward intermediate WBC between 450 m and 1800 m flowing into the southern Maluku Sea through the western channel (Fig. 1b), and a weak northward flow in the central and eastern channel recorded by the moorings M02, M03, and M00 (Fig. 1e−h, Supplementary Fig. 1). Volume flux estimates and numerical model results presented later suggest that this northward flow is a return flow fed by the boundary flow. The currents at M01 are constrained by the coasts, with the maximum mean southward along strait velocity (ASV) over 5 cm s^−1^ at the core depth of 1000 m. This intermediate flow along the western boundary has been indicated by historically current measurements at 750 m and 1250 m at a location very close to M01^22^. But the western intensification of the channel currents was not recognized and mentioned. The fact that the core depth of this current is deeper than the threshold depth of the Makassar Strait underlines the uniqueness and importance of this intermediate WBC in connecting the Pacific and Indian Ocean intermediate-depth circulation.

Destination and origin of the intermediate WBC. We suggest that the Maluku Channel intermediate WBC should reach the Lifamatola Passage and continue southward, since the latter is the only deep opening to the southern Indonesian seas. The LF mooring observations indeed show that the mean currents below the depth of 450 m are southward into the Seram and Banda Seas above the 95% confidence level (Fig. 1c–d).

**Fig. 2:**
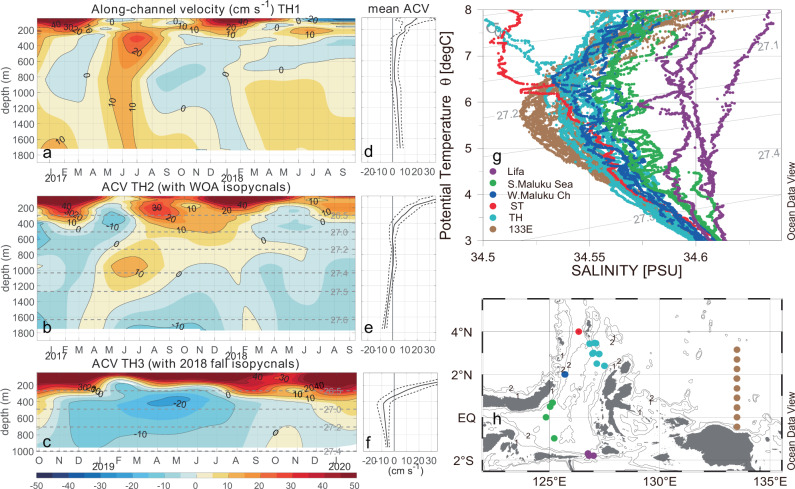
TH channel velocity and Maluku Sea water masses properties. The 120-day low-passed daily along-channel velocity (ACV) time series (**a**–**c**) and their mean profiles (**d**–**f**) of moored current meter measurements in the TH Channel, showing intrusions of Pacific intermediate waters into the Maluku Sea through the northern TH Channel. The ACV is defined as 67° clockwise from due north perpendicular to a section from Talaud to Halmahera Islands. Dash lines mark the standard errors of the means at 95% significance. The isopycnal depths calculated from the temperature and salinity profiles of the World Ocean Atlas 2013 version 2 and of the fall 2018 cruise are marked in (**b**) and (**c**). **g** Potential temperature-salinity relations of the intermediate water masses in the TH Channel and the Maluku Channel, showing the AAIW salinity minimum increasing from the Pacific western boundary (133°E sections) through the TH Channel to the western Maluku Channel. The only one CTD profile in the Sangihe-Talaud Channel (red dot in **g** and **h**) is also shown to be similar to the western Maluku Channel profiles with the AAIW properties. Dots in (**h**) are the CTD stations of (**g**), with the colors used to represent the straits or regions.

We further suggest that the Maluku Channel intermediate WBC comes from the inflow through the northern TH Channel (Fig. 2). Although the TH1 mooring shows mean intermediate currents flowing into the Pacific Ocean from the Maluku Sea, the TH2 mooring has recorded intrusions of the Pacific waters into the Maluku Sea, except during the summer of 2017. The mooring data at TH3 after 2018 clearly show currents flowing into the Maluku Sea through the northern TH Channel between 300 m and 1000 m. The suggestions, then, are that the inflows through the mooring sites of TH2 and TH3 feed into the intermediate WBC in the western Maluku Channel. Due to lack of direct measurements, the currents through the Sangihe-Talaud Strait are not known at present.

The relation of the potential temperature *θ* and the salinity *S* is used to identify the water mass of the AAIW in the equatorial western Pacific. All of the hydrographic data were collected using a SBE 911 plus CTD instrument onboard of the R/V Baruna Jaya VIII. The *θ–S* relations of the water masses in the TH channel and in the western Maluku Channel during the mooring maintenance cruises are similar to that of the AAIW in the 133°E section in the equatorial western Pacific Ocean, with a salinity minimum around the isopycnal *σ*_*θ*_ = 27.2 kg m^−3^ (Fig. 2g). The *θ–S* relation in the Sangihe-Talaud Strait below *σ*_*θ*_ = 27.2 kg m^−3^ is similar to that in the western Maluku Channel. The salinity minimum in the western Maluku Channel is slightly larger than that in the TH Channel, which is in turn larger than that in the 133°E section, consistent with the intrusion of the AAIW from the western Pacific into the western Maluku Channel through the TH Channel subject to the strong mixing at the entrance and inside the Indonesian seas^23^. The water mass analysis suggests that the AAIW reaches the Lifamatola Passage during the mooring measurements (Fig. 2g), with the salinity minimum largely eroded by the strong mixing inside the Maluku Sea. Considering that the southward flow through the Lifamatola Passage can be elevated above the 1000 m threshold of the ITF exits by the strong mixing inside the Indonesian seas^19–21^, this intermediate-depth throughflow is suggested to carry the intermediate waters from the South Pacific (Supplementary Figs. 2 and 3) and to the tropical southeastern Indian Ocean through the eastern Indonesian seas.

**Fig. 3:**
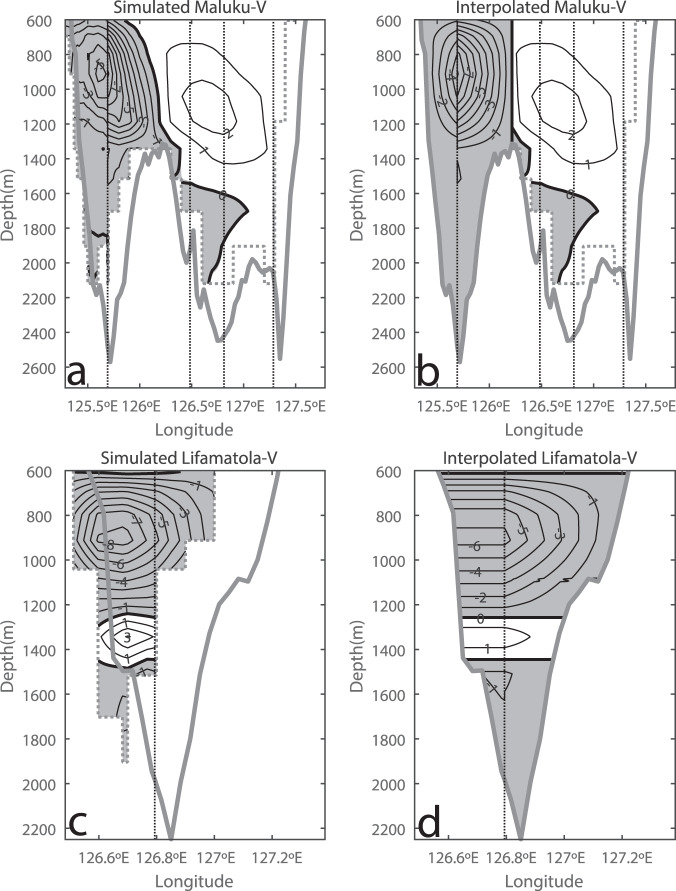
Current porfiles in the Maluku Channel and the Lifamatola Passage. **a** OFES model simulated mean currents, through the Maluku Channel normal to a coast-to-coast section along 2°N, and (**b**) the interpolated WBC from simulated M01 data using the nonslip boundary condition on both sides of the western Maluku Channel, respectively; **c** simulated mean currents through the Lifamatola Passage normal to a shortest coast-to coast section, and (**d**) the interpolated currents using the freeslip and nonslip conditions on the western and eastern coasts, respectively, showing good approximation of the interpolated to the simulated currents. Unit is cm s^−1^. The simulated and interpolated transports between 600 m and 1200 m are 2.08 Sv and 1.75 Sv, respectively, for the WBC (**a**, **b**), whereas 1.25 Sv and 0.96 Sv, respectively, through the LF Passage (**c**, **d**). The dash stairs stand for model topography. The thick contours in the Maluku Channel section and the Lifamatola Passage section are the water depths from the ETOPO2v2 database.

Due to the small numbers of moorings, especially in the Maluku Channel, the estimates of the transports of the WBC and the throughflow are subject to large uncertainties, due to the use of different interpolation schemes and boundary conditions. Based on the Ocean General Circulation Model for the Earth Simulator (OFES) (Fig. 3), the intermediate WBC is suggested to be confined within the western channel. The calculation of the WBC transport using the mooring data between 450 m and 1800 m based on the nonslip boundary condition on both sides of the western Maluku Channel suggests a southward transport of 2.28 Sv (1 Sv = 10^6^ m^3^ s^−1^), larger than the southward transport of 1.36 Sv in the Lifamatola Passage in the same depth range. In comparison, the Makassar Strait mean transport between 450 m and 760 m depths calculated from mooring data is about 1.7 Sv, assuming the nonslip condition, which is the mixture of the NPIW and lower SPTW from the North Equatorial Subsurface Current^24^. The AAIW is too deep to enter the Makassar Strait in general. The transports through the Lifamatola Passage were estimated across a section of the shortest distance between the coasts using freeslip and nonslip boundary conditions on the western and eastern coast, respectively, to simulate the western intensification of the currents (Fig. 3). The rest of the WBC transport is suggested to circulate back to the Pacific Ocean through the central and eastern Maluku Channel.

We used the OFES model simulation to estimate the uncertainty of the WBC transports. The WBC transports between 600 m and 1200 m based on the simulated and the interpolated M01 velocity in the model are calculated to be 2.08 Sv and 1.75 Sv, respectively, in the western Maluku Channel (Fig. 3a, b). The simulated and interpolated LF transports are 1.25 Sv and 0.96 Sv, respectively (Fig. 3c, d). The comparisons suggest that the uncertainty of the transports estimated from the interpolation methods is smaller than the simulated mean transports.

It is worth mentioning that our LF mooring is located upstream and to the west of the van Aken mooring^20^, which shows deep overflow into the abyssal Seram and Banda Seas along the thalweg in the southeastern direction and weak northwestward currents above 1250 m. The difference between the two observations can be explained by the precipitous descending of temperature and salinity contours deeper than 1500 m from upstream down the slope of the Lifamatola threshold (Supplementary Fig. 4), suggesting that the overflow is partially fed by the southward transport in the intermediate layer. In addition, the channel is wider downstream of the saddle point and flow reversals often occur above the bottom overflow experiencing strong turbulence and entrainment^19–21^, which suggest potential existence of horizontal recirculation associated with the western intensified currents and the reversal currents above the bottom overflow.

**Fig. 4:**
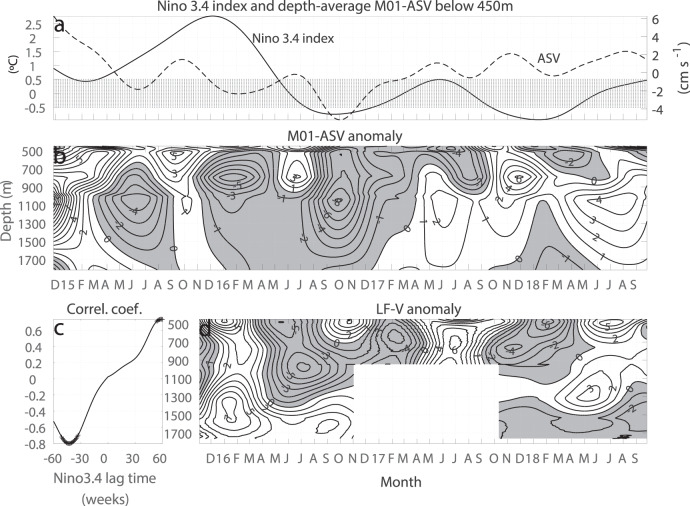
Interannual variations. Maluku Sea intermediate WBC anomalies (**a**, **b**), showing significant lead and lag correlations with Niño3.4 index (**c**), and co-variability with the currents through the Lifamatola Passage (**d**). The black Crosses in (**c**) represent the correlation coefficient above 95% significance level. Units are cm s^−1^ for velocity and °C for Niño3.4 index. Contour interval is 1 cm s^−1^.

Interannual variations. The intermediate WBC in the Maluku Channel is subject to strong interannual variations associated with the 2015/2016 strong El Niño (Fig. 4). The mooring data have shown that the WBC speeds increased since the summer of 2015, which persisted until early 2017. Lag correlation analysis suggests that the WBC lags the Niño3.4 index by about 40 weeks, which can be explained by the vertical propagation of equatorial and off-equatorial Rossby waves from the eastern and central equatorial Pacific forced by the winds and wind stress curl, respectively^25,26^.

The correlation calculation also suggests that the WBC leads the Niño3.4 index by a year or so. Since the Maluku Channel is situated on the equatorial Kelvin wave guide, this leading relation may suggest the influence from the Indian Ocean Dipole at the 1-year lead^27–29^.

The LF mooring intermediate currents have shown similar interannual variations as the Maluku Channel WBC, with a delay of about 3 months. Due to the short time series of the LF mooring, the lag correlation coefficient could not pass the statistical significance test. More studies are needed to understand the interannual variations of the Maluku Sea intermediate circulation associated with the El Niño and the Southern Oscillation.

## Discussion

**Fig. 5:**
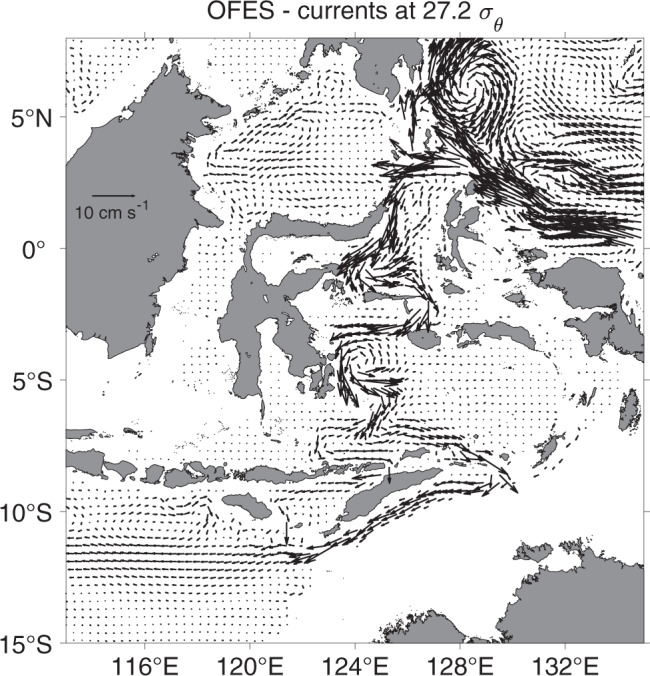
Intermediate throughflow in the OFES. Distributions of mean currents on the 27.2 σ_θ_ isopycnal layer, based on the OFES model simulation during 2000–2018, showing the Maluku WBC originating from the NGCUC in the western equatorial Pacific Ocean. The NGCUC and WBC carries the subducted AAIW from the South Pacific Ocean to the southeastern tropical Indian Ocean through the eastern Indonesian seas.

The mean currents of OFES simulation^30^ during 2000 through 2018 have simulated successfully the intermediate throughflow from the equatorial western Pacific through the western Maluku Sea and the Lifamatola Passage into the Seram and Banda Seas (Fig. 5). A weak cyclonic gyre returns part of the WBC transport back to the Pacific Ocean through the eastern Maluku Channel, just as the mooring data of M02, M03, and M00 have indicated. Our mooring observations have detected a previous undocumented branch of the ITF that connects the intermediate ocean circulation in the western Pacific and Indian Ocean through the Indonesian seas. This branch of the ITF constitutes a previously unknown path of the ocean ventilation from the South Pacific north of the Antarctic Circumpolar Current to the southeastern tropical Indian Ocean (Supplementary Fig. 2), providing a conduit for the subducted Pacific intermediate waters to come back to the surface of the ocean through the wind-driven overturn of the Indian Ocean. Traditionally, the intermediate waters like AAIW are believed to return to the sea surface through the very slow diffusive process in the tropical and subtropical Pacific. More studies are needed to investigate the role of the intermediate WBC and ITF in the ocean intermediate circulation and in global overturn, which will improve our understanding of global ocean heat and CO_2_ storages and fresh water balances associated with climate changes.

## Methods

### Moored ADCP measurements

The bin size and the time interval of the ADCP sampling are 8 m and 1 h, respectively. The RCM sampling interval is 1 h. A few bins of missing value are filled with linear interpolation in the vertical. The ADCP and RCM data are interpolated onto a 1 m vertical grid and are filtered with the Thompson filter^31^ to remove tidal signals from the hourly velocity series before averaged into daily means. In this study, we focus on the mean currents and interannual variability. A 4th-order Butterworth low-pass filter with a 120-day cutoff period is applied to suppress the high-frequency oscillations including intraseasonal variability.

### OFES Model

The OFES^30^ employs a horizontal resolution of 0.1° longitude by 0.1° latitude with 54 uneven *z* levels in the vertical. The hindcast simulation is forced by the NCEP/NCAR daily winds and monthly heat flux and precipitation during 2000 through 2018. In addition, the sea surface salinity is restored to the monthly climatology of the World Ocean Atlas 1998 with the nudging time scale of 6 days, to represent the effects of runoffs and the evaporation minus precipitation.

### Other observational data

The two arc-Minute Gridded Global Relief Data ETOPO2v2 data of the U.S. National Geophysical Data Center are used to calculate the transports in the western Maluku Channel and the Lifamatola Passage, according to comparisons with the echo sounder measurements.

The Niño3.4 index data are averaged sea surface temperature (SST) anomalies in the box 170°W–120°W, 5°S–5°N, using Reynolds OIv2 SST analysis data. The anomalies are calculated relative to a monthly climatological seasonal cycle based on the years 1982–2005. The monthly climatology is linearly interpolated to determine weekly anomalies. Spatial averaging of the gridded analysis was weighted by surface area.

The gridded Argo data used in this study include salinity and temperature profiles on a 1° longitude × 1° latitude horizontal grid and in 58 vertical levels from 2.5 m to 1975 m in monthly archives spanning the time period from January 2004 to December 2016. The absolute geostrophic currents (AGCs) are calculated from the monthly gridded Argo profiles between 800 m and 2000 m using the P-vector method^32^. The AGCs above the 800 m are calculated using the dynamic height calculation, referenced to the AGCs at 800 m. In this study, we use the average AGCs from January 2004 to December 2016 to describe the AAIW movement.

## Supplementary information

Supplementary Information

## Data Availability

The processed mooring data are available at the website http://itf.qdio.ac.cn/xzlxz/. The monthly OFES data can be accessed at http://apdrc.soest.hawaii.edu/datadoc/ofes/ofes.php. The ETOPO2v2 data can be downloaded at https://www.ngdc.noaa.gov/mgg/global/etopo2.html. The Niño3.4 index data are downloaded from the NOAA webpage https://stateoftheocean.osmc.noaa.gov/sur/pac/nino34.php. The gridded Argo data are obtained from the Argo website http://www.argo.ucsd.edu.

## Code availability

The codes used to process and analyze the data are available at the website http://itf.qdio.ac.cn/xzlxz/.
