# Supplementary material for: A Maluku Sea intermediate western boundary current connecting Pacific Ocean circulation to the Indonesian Throughflow
Source: Nat Commun. 2022 Apr 19;13:2093. doi: 10.1038/s41467-022-29617-6 (PMC9018790; doi:10.1038/s41467-022-29617-6)
Supplement: Supplementary file 1 — Supplementary Information [file 41467_2022_29617_MOESM1_ESM.pdf]

Supplementary Information for

A Maluku Sea intermediate western boundary current connecting Pacific Ocean circulation to the Indonesian Throughflow

Dongliang Yuan[1,2,3,4,5,*], Xueli Yin[1,3,5,†], Xiang Li[1,3,†], Corry Corvianawatie[1,3,5,6], Zheng Wang[1,3], Yao Li[1,3], Ya Yang[1,2,3,4,5], Xiaoyue Hu[1,3,5], Jing Wang[1,3,5], Shuwen Tan[1,3,5], Dewi Surinati[6], Adi Purwandana[6], Adhitya Kusuma Wardana[6], Mochamad Furqon Azis Ismail[6], Asep Sandra Budiman[6], Ahmad Bayhaqi[6], Praditya Avianto[6], Priyadi Dwi Santoso[6], Edi Kusmanto[6], Dirhamsyah[6], Zainal Arifin[6], Larry J. Pratt[7]

[1]Key Laboratory of Ocean Circulation and Waves, and Center for Ocean Mega-Science, Institute of Oceanology, Chinese Academy of Sciences, 7 Nanhai Road, Qingdao, China
[2]Key Laboratory of Marine Science and Numerical Modeling, First Institute of Oceanography, Ministry of Natural Resources, 6 Xianxialing Road, Laoshan District, Qingdao, China
Affiliation 1 and Affiliation 2 share the first position
[3]Pilot National Laboratory for Marine Science and Technology (Qingdao), Qingdao, China
[4]Shandong Key Laboratory of Marine Science and Numerical Modeling, 6 Xianxialing Road, Laoshan District, Qingdao, China
[5]University of Chinese Academy of Sciences, Beijing, China
[6]Research Center for Oceanography–National Research and Innovation Agency (RCO-BRIN), Jakarta, Indonesia
[7]Department of Physical Oceanography, Woods Hole Oceanographic Institution, Woods Hole, Massachusetts

*Correspondence to: dyuan@fio.org.cn
Address: First Institute of Oceanography, Ministry of Natural Resources, 6 Xianxialing Road, Laoshan District, Qingdao 266061, China
†Co-first author: Institute of Oceanology, Chinese Academy of Sciences, 7 Nanhai Road, Qingdao, 266071, China
These authors contributed equally: D. Yuan, X. Yin, and X. Li

Inventory of this file

Supplementary Methods: 1

Supplementary figures: 5

Supplementary References: 2

Supplementary Methods

The correlation coefficients are calculated as:

$$r = \frac{\sum_{i=1}^{n}(X_i - \bar{X})(Y_i - \bar{Y})}{\sqrt{\sum_{i=1}^{n}(X_i - \bar{X})^2}\sqrt{\sum_{i=1}^{n}(Y_i - \bar{Y})^2}} \qquad (1)$$

where X and Y are time series of two variables and n is the length of the series.

Because the M01-ASV and the Nino 3.4 index series has been low-passed filtered with the cutoff period at 26 weeks, the number of degrees of freedom of the filtered time series is expect to be far below the length of the series. The Monte Carlo method was used to compute the critical values of significant correlation coefficients, following the steps below (supplementary reference 1):

(1) Given the length of the sequence, generate two red noise sequences (supplementary reference 2), and compute the correlation coefficient of them after low-passed filtering.

(2) A series of correlation coefficients were obtained after repeating the aforementioned steps 5,000 times, which are sorted in an ascending order. The 4750th (5000x95%) number in the series is regarded as the correlation coefficient thresholds at 95% significant level.

(3) Forty correlation coefficient thresholds were obtained after repeating the above steps 40 times, and the mean of them was regarded as the reliability thresholds of the critical correlation coefficients at 95% significant level.

(4) Change the length of the random sequence in Step (1) and carry the Steps (1)-(3) to obtain the correlation coefficient thresholds at different lengths of the input series, which are used to test the significance of the lag correlations of shortened time series. In our research, the smallest length of the input series used to calculate the lag correlation coefficients are 141, in contrast to the full length of 201 weekly data.

Based on the obtained correlation coefficient thresholds at 95% significant level, the degrees of freedom were inferred to be about 6 to 7 by checking the Table of critical values of correlation coefficient.

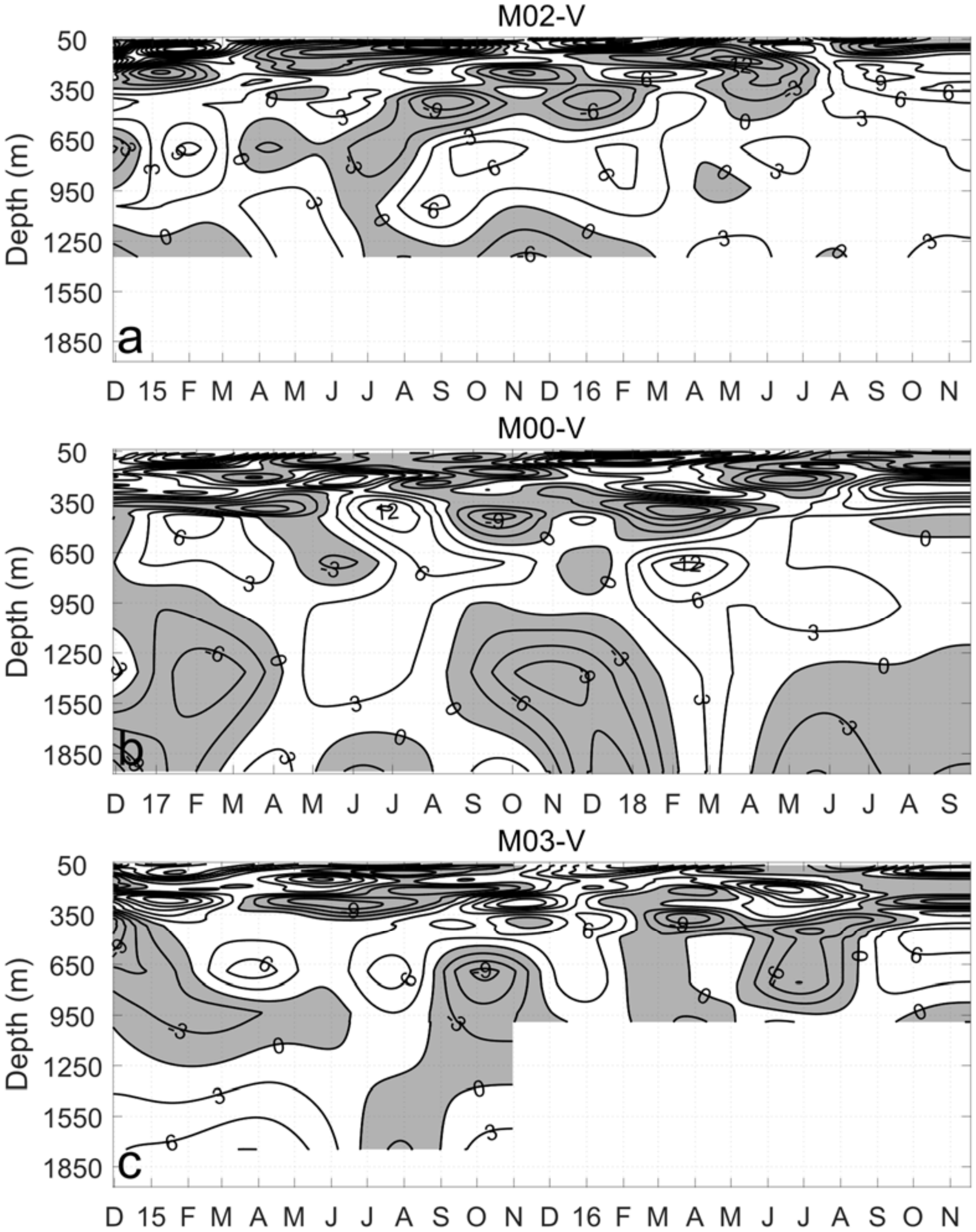

Supplementary Fig. 1 | Meridional velocity in the eastern Maluku Channel. 120-day low-passed meridional velocity of the mooring M02, M00, and M03 (a-c), showing the weak return flows in the central and eastern channel. Unit is cm s^{-1}. Contour interval is 3 cm s^{-1}.

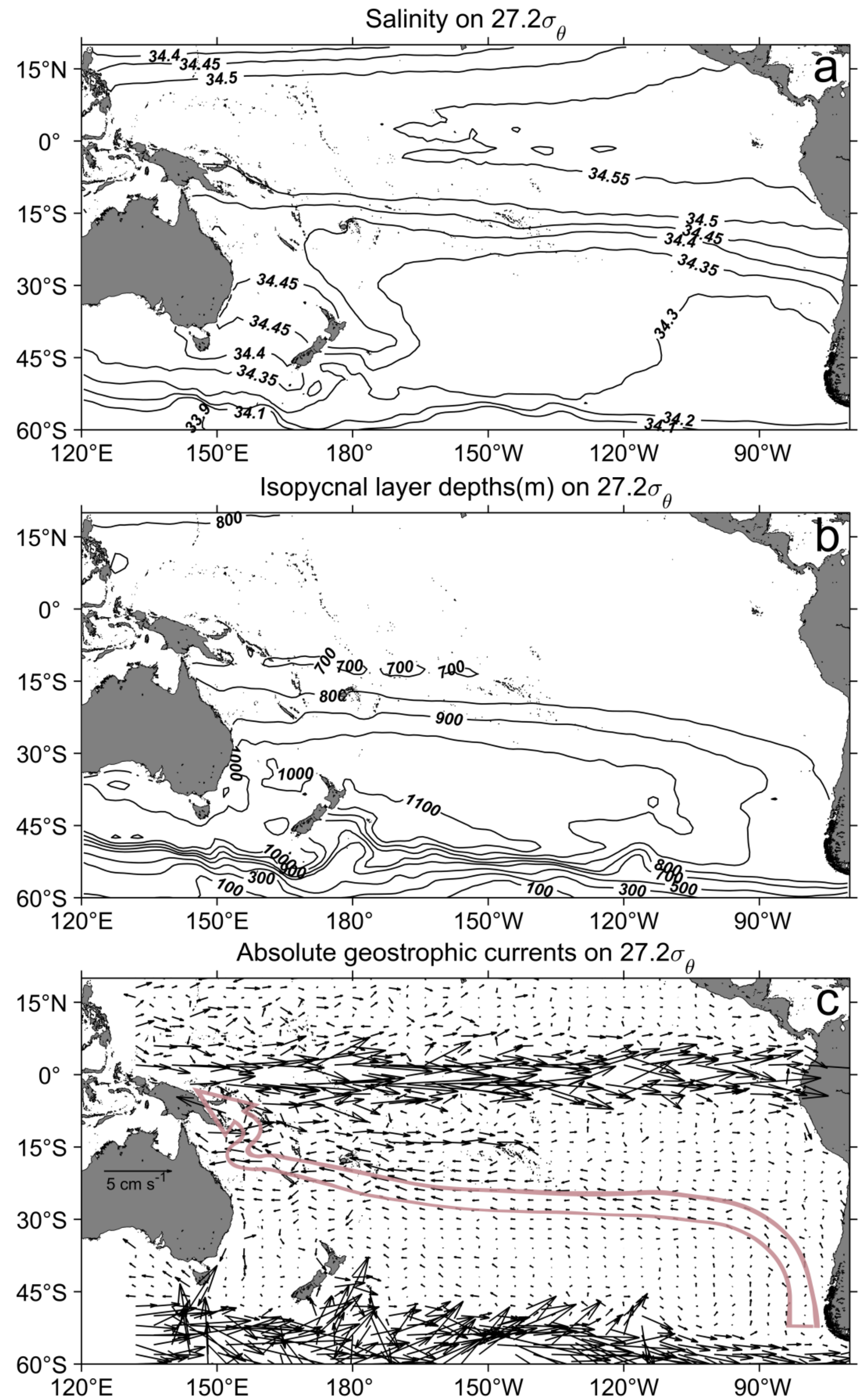

Supplementary Fig. 2 | Argo geostrophic currents and properties on the 27.2σθ isopycnal layer. Distributions of salinity (a) and isopycnal layer depths (b), showing the isopycnal contours channel lowest salinity waters from the southeastern Pacific Ocean into the deep part of the South Equatorial Current flowing westward into the western boundary current, a.k.a. the NGCUC. The flow pattern is supported by the P-vector absolute geostrophic currents on the 27.2σθ isopycnal layer (c), based

on the mean Argo data during 2004-2016. Units are psu for salinity, m for isopycnal layer depths, and cm s^{-1} for the absolute geostrophic currents. The big arrow in (c) mark the movement of the AAIW.

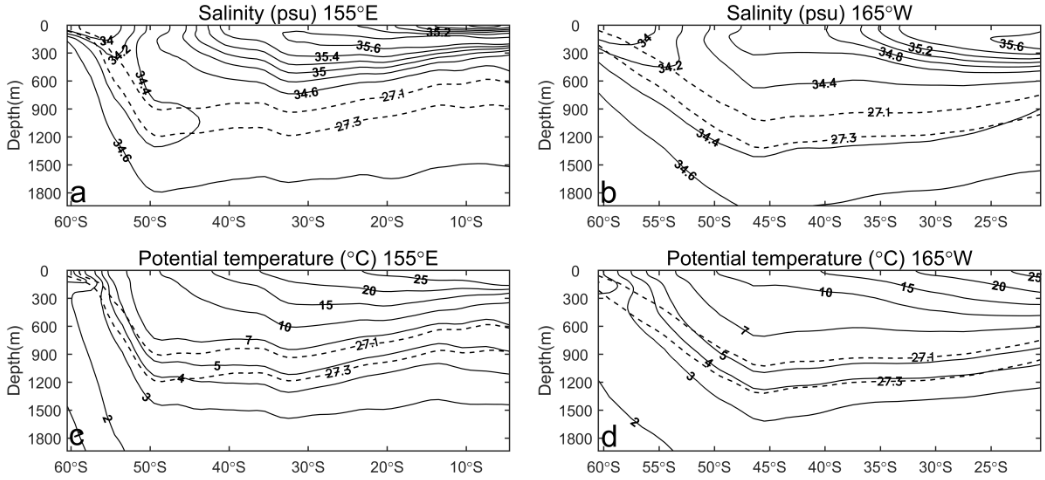

Supplementary Fig. 3 | Argo salinity (solid contours) distributions in the Coral-Tasman Sea meridional section (a) and in the central South Pacific Ocean meridional section (b), and potential temperature distributions (solid contours) in the Coral-Tasman Sea meridional section (c) and in the central South Pacific Ocean meridional section (d), showing outcropping of the 27.1 σ_θ-27.3 σ_θ isopycnals (dash contours) in the Sub-Antarctic Front associated with the subduction of the AAIW. Units are psu for salinity and ℃ for potential temperature. Salinity and potential temperature distributions are based on the mean Argo data during 2004-2016.

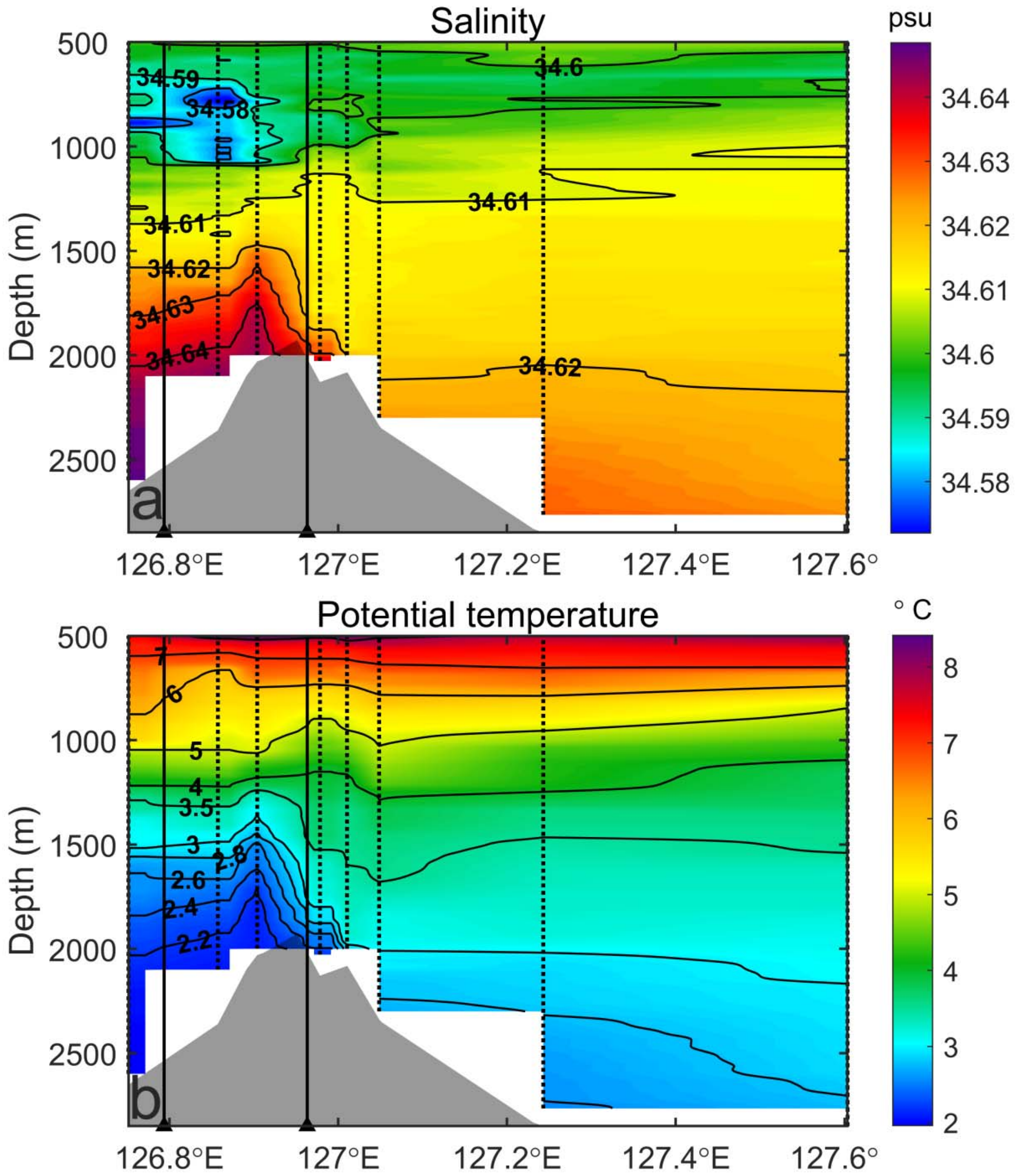

Supplementary Fig. 4 | Lifamatola Passage bottom overflow. (a) Salinity and (b) potential temperature distributions (color and contours) along the thalweg of the Lifamatola valley during the October 2017 cruise, showing descending of salinity and potential temperature contours downstream of the saddle point. Two black triangles with vertical black lines above from the west to the east represent the location of the LF mooring and the historical van Aken's mooring, respectively. Note that the LF mooring was deployed a little to the west of the thalweg at a depth of about 2150 m. The topography is based on the shipboard echo sounder measurements. The black dot lines mark the locations of the CTD stations and their corresponding measurement depths. Units are psu for salinity and ℃ for potential temperature.

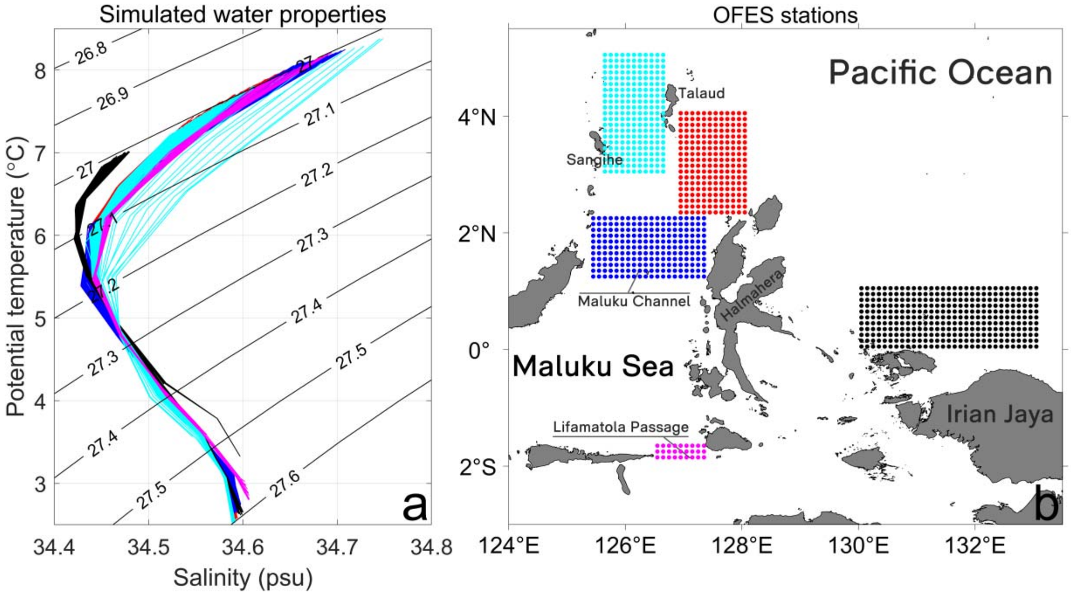

Supplementary Fig. 5 | Water masses properties in the intermediate western Pacific Ocean and Maluku Sea. Potential temperature-salinity relation (a) of the intermediate water masses (b) in the TH Channel, Maluku Channel, Sangihe-Talaud Channel, Lifamatola Passage and in the western Pacific Ocean based on OFES outputs, showing the salinity minimum of the AAIW at around 27.1σ_θ-27.2σ_θ. Dots in (b) are the locations of profiles in (a) of corresponding colors.

Supplementary References

1. Li,Z. , Song, Y. et al. Interdecadal correlation of solar activity with Tibetan Plateau snow depth and winter atmospheric circulation in East Asia. *Sciences in Cold and Arid Regions*. **8** (006), 524-535 (2016).
2. H. Zhivomirov. A Method for Colored Noise Generation. Romanian Journal of Acoustics and Vibration, ISSN: 1584-7284, Vol. XV, No. 1, pp. 14-19, (2018).
